# ﻿Description of a new *Promastobranchus* species (Annelida, Capitellidae) from Chinese coasts, with molecular evidence for intraspecific variation in the number of thoracic chaetigers

**DOI:** 10.3897/zookeys.1174.106624

**Published:** 2023-08-08

**Authors:** Jun-Hui Lin, María E. García-Garza, Jian-Feng Mou, He-Shan Lin

**Affiliations:** 1 Third Institute of Oceanography, Ministry of Natural Resources, 178 Daxue Road, Xiamen 361005, China Third Institute of Oceanography, Ministry of Natural Resources Xiamen China; 2 Universidad Autónoma de Nuevo León, Facultad de Ciencias Biológicas, Laboratorio de Biosistemática, Apartado Postal 5 “F”, San Nicolás de los Garza, Nuevo León, Mexico Universidad Autónoma de Nuevo León San Nicolás de los Garza Mexico

**Keywords:** Mitochondrial markers, nuclear markers, polychaete, South China Sea, taxonomy

## Abstract

*Promastobranchus* Gallardo, 1968 is a small genus in the polychaete family Capitellidae, and the available records are largely reported from the Indo-West Pacific region. Although Gallardo (1968) and Green (2002) noted that *Promastobranchus* species had intraspecific variation in the number of thoracic chaetigers when they described the two previously known species, this variation has not been corroborated using molecular evidence. In this study, a new *Promastobranchus* species, *Promastobranchusvariabilis***sp. nov.**, is described based on 29 specimens collected from the Beibu Gulf, South China Sea. The new species is mainly characterized by having a tessellated epithelium in the anterior thorax, nine teeth in three rows above the main fang in the abdominal hooks, four pairs of genital pores located on the intersegmental grooves between chaetigers 9 and 13, and its unique methyl green staining pattern. Comparisons of multiple gene markers (16S, 18S, 28S, and H3) revealed no genetic divergence (K2P < 0.003) among these type specimens with 9–13 thoracic chaetigers. In other words, the new species exhibited morphological variability in the number of thoracic chaetigers during ontogeny, and this character was unsuitable to differentiate *Promastobranchus* species as the ranges overlap among *Promastobranchus* species. This is the third *Promastobranchus* species known in the world, and it is now recorded along the Fujian coast.

## ﻿Introduction

Members of the polychaete family Capitellidae are commonly encountered components of marine infaunal communities and are especially abundant in organically enriched sediments (Blake 2000; Magalhães and Blake 2020). To date, the family contains more than 200 described species in 41 genera (Read and Fauchald 2023). Of these genera, *Promastobranchus* Gallardo, 1968 is a small genus with only two described species worldwide. *Promastobranchus* is a unique genus, and it can be readily distinguished from other capitellid genera in bearing relatively long capillary chaetae in the notopodia throughout the abdomen, as well as the relatively long prechaetal region (Green 2002; Magalhães and Blake 2020). According to the available records, *Promastobranchus* appears to be endemic to the Indo-West Pacific region. Type localities of the two known species are located in this region: *Promastobranchushuloti* Gallardo, 1968 from nearshore waters off southern Vietnam and *Promastobranchusorbiculatus* Green, 2002 from the Thailand part of the Andaman Sea. Other records of *Promastobranchus* were also reported from this region, such as Natuna Islands (Al-Hakim and Glasby 2004), Sulawesi Island (Lin et al. 2018), and northern Australian (data from Ocean Biodiversity Information System, https://www.obis.org).

The number of thoracic chaetigers is an important diagnostic character used to differentiate capitellid genera (Blake 2000). The majority of species in Capitellidae bear a fixed number of thoracic chaetigers without intraspecific variation. Species of *Notomastus* Sars, 1851 have 11 thoracic chaetigers while those of *Mediomastus* Hartman, 1944 have 10. However, *Leiocapitella* Hartman, 1947 has interspecific but not intraspecific variation in the number of thoracic chaetigers (12–16). More specially, *Promastobranchus*, together with *Scyphoproctus* Gravier, 1904, have been reported to have intraspecific variation in the number of thoracic chaetigers. *Promastobranchushuloti* was reported to have 12 or 13 thoracic chaetigers (Gallardo 1968). Green (2002) described *P.orbiculatus* based on specimens with 9–12 thoracic chaetigers with capillary chaetae in both rami, and these specimens possessed an identical dental formula of hooks. Nevertheless, morphological variability had not been corroborated using molecular evidence until this study. In recent samplings, 29 *Promastobranchus* specimens with 9–13 thoracic chaetigers were collected from the Beibu Gulf, South China Sea. Using multiple gene markers of the newly collected specimens, we attempted to verify whether these specimens belong to an identical species or whether there was hidden diversity. The results indicate that these specimens belong to an identical species without genetic divergence and that the species is new to science, *P.variabilis* sp. nov. The new species bears a variable number of thoracic chaetigers (9–13), a character not useful for differentiation among *Promastobranchus* species. This is the third species in the genus *Promastobranchus* worldwide. The new species also occurs along the Fujian coast, approximately 1100 km northeast of its type locality, based on genetic comparison. The phylogenetic position of *Promastobranchus* and its sister genera within the family will be evaluated when molecular data of its closest related genera (e.g., *Scyphoproctus*) are available.

## ﻿Materials and methods

### ﻿Field sampling

**Figure 1.**
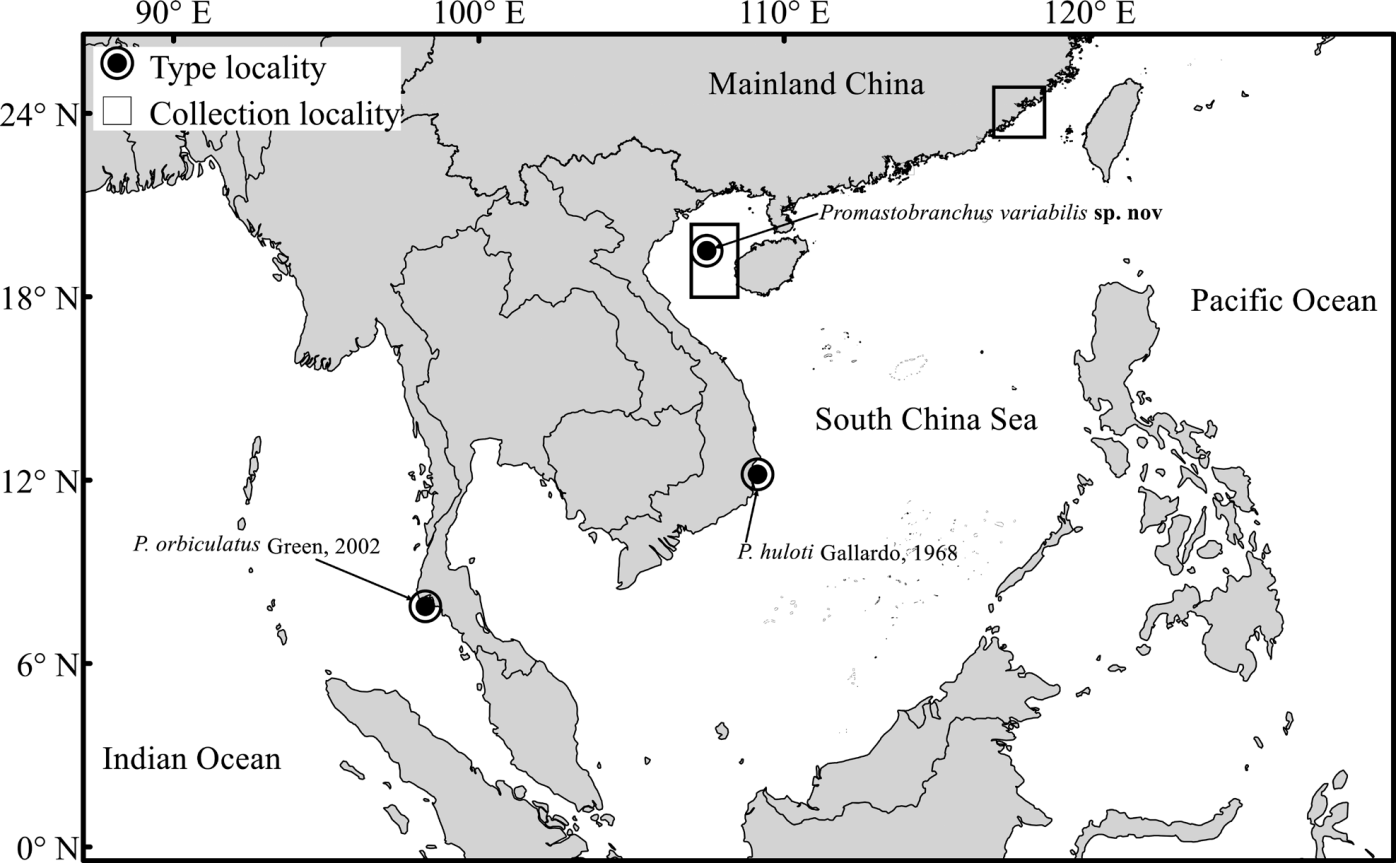
Type localities of the three *Promastobranchus* species (circles) and two sampling localities (squares) included in this study

Sampling was conducted at two localities (Beibu Gulf, South China Sea and coastal waters of Fujian Province) along south Chinese coast during 2020-2022 (Fig. 1). Sediment samples were collected using a grab sampler and subsequently washed through a 0.5 mm sieve in the field. The specimens retained in the sieve were directly preserved in 90% ethanol.

### ﻿Morphological observation

In the lab, specimens of the target species were picked out of the samples and examined under a Leica MZ95 stereoscope. Detailed information on the examined specimens is shown in Table 1. Light photographs were taken under a Leica M205A stereoscope equipped with a DFC 550 digital camera. The structure of abdominal hooks was observed under a Leica DM6B microscope using oil emersion (×100). Observations were performed on a scanning electron microscope (TESCAN MIRA) at the Third Institute of Oceanography, and the methyl green staining pattern (MGSP) was used to identify the distribution of glandular areas, both as delineated by Lin et al. (2019). The type material and additional material examined in this study were deposited at the Third Institute of Oceanography, Ministry of Natural Resources, Xiamen, China (**TIO**, MNR).

**Table 1.** Information on the sampling and body size for the examined specimens of *Promastobranchusvariabilis* sp. nov. Abbreviations: NoTC: number of thoracic chaetigers; N: number of individuals; TC: total chaetigers; TL: total length; BW: body width.

Location	Sta.	Lat. (N)	Long. (E)	Date yyyymmdd	NoTC	N	TC	TL mm	BW mm	Habitat	Type	Voucher	16S	18S	28S	H3
Beibu Gulf SCS	S31	19.65	107.698	20210523	11	1	71	35.99	1.23	65 m; mud	holotype	Poly139	OR000758	OR000744	OR000731	OQ999658
					13	1	15	8.28	1.3	65 m; mud	paratype	Poly140	OR000759	OR000745	OR000732	OQ999659
	S42	19.25	108.098	20210524	11	4	18–35	5.04–19.78	0.54–0.94	59 m; muddy sand with shell fragment	paratype	Poly141	OR000760	OR000746	OR000733	OQ999660
	S44	19.25	108.498	20210524	9	3	17–27	2.68–5.30	0.47–0.68	36 m; muddy sand	paratype	Poly142	OR000761	OR000747	OR000734	OQ999661
					10	5	16–23	3.20–5.75	0.45–0.77	36 m; muddy sand	paratype	Poly143	OR000762	OR000748	OR000735	OQ999662
					11	6	17–22	3.96–8.80	0.66–0.94	36 m; muddy sand	paratype	Poly144	OR000763	OR000749	OR000736	OQ999663
	S51	18.85	108.498	20210526	10	4	17–27	3.08–4.78	0.62–0.86	15 m; sand, muddy sand	paratype	Poly145	OR000764	OR000750	OR000737	OQ999664
					11	3	18–27	4.11–8.49	0.65–0.81	15 m; sand, muddy sand	paratype	Poly146	–	OR000751	OR000738	OQ999665
					12	1	19	7.10	0.96	15 m; sand, muddy sand	paratype	Poly147	OR000765	OR000752	OR000739	OQ999666
	S14	20.456	108.495	20221127	12	1	34	19.12	1.52	55 m; muddy sand	paratype	Poly148	OR000766	OR000753	–	OQ999667
Fujian coast	XM08	24.5	118.2167	20211029	10–11	2	20–26	4.13–6.69	0.44–0.55	17 m; muddy sand	nontype	Poly149	–	OR000754	OR000740	OQ999668
	Q36	24.5126	118.207	20220105	9–11	6	19–43	4.77–11.76	0.42–0.70	10 m; muddy sand	nontype	Poly150	–	OR000755	OR000741	OQ999669
	D15	24.5025	118.2263	20201211	9–10	3	18–36	6.02–10.80	0.59–0.75	15 m; muddy sand	nontype	Poly151	OR000767	OR000756	OR000742	OQ999670
	HX2	23.8708	117.5182	20221221	11	1	14	3.40	0.75	10 m; mud	nontype	Poly152	OR000768	OR000757	OR000743	OQ999671

### ﻿DNA extraction, PCR amplification, and sequencing

The total genomic DNA was extracted from organisms using Transgen Micro Genomic DNA EE 181 Kit (Transgen, Beijing, China) following the protocol provided by the manufacturer. Polymerase chain reactions (PCRs) were conducted to amplify partial sequences of mitochondrial (16S) and nuclear (18S, 28S, H3) genes using primer sets and thermal cycling conditions as shown in Table 2. The 50-μL PCR mixtures contained 25 μL of 2X PCR Mix (Vazyme, Nanjing, China), 2 μL of DNA template, 2 μL of each primer, and 19 μL of deionized water. Then, the 5-μL PCR products were subsequently checked using 1% agarose gel electrophoresis. Sequencing of the successful products was performed in both directions at the Sangon Co. (Shanghai, China) with an ABI 3730XL DNA analyzer (Applied Biosystems). Both forward and reverse strands of sequences were manually assembled into a consensus sequence using DNAMAN software (Lynnon Biosoft, Quebec, Canada).

**Table 2.** List of primer sets used for PCR and sequencing in this study.

Gene	Primer	Sequence (5′ to 3′)	Reference	Thermal cycling conditions
16S	16SarL	CGCCTGTTTATCAAAAACAT	Palumbi 1996	95 °C/3 min; 35 × (95 °C/40 s, 47 °C/40 s; 72 °C/1 min); 72 °C/7 min
	16SbrH	CCGGTCTGAACTCAGATCACGT	Palumbi 1996	
	Ann16S	GCGGTATCCTGACCGTRCWAAGGTA	Sjölin et al. 2005	
	16SbrH	CCGGTCTGAACTCAGATCACGT	Palumbi 1996	
18S	18SA	AYCTGGTTGATCCTGCCAGT	Medlin et al. 1988	95 °C/3 min; 35 × (94 °C/45 s, 55 °C/45 s; 72 °C/2 min); 72 °C/10 min
	18SB	ACCTTGTTACGACTTTTACTTCCTC	Nygren and Sundberg 2003	
	620F	TAAAGYTGYTGCAGTTAAA	Nygren and Sundberg 2003	
	1324R	CGGCCATGCACCACC	Cohen et al. 1998	
28S	Po28F1	TAAGCGGAGGAAAAGAAAC	Struck et al. 2006	95 °C/3 min; 40 × (95 °C/30 s, 55 °C/40 s; 72 °C/75 s); 72 °C/7 min
	Po28R4	GTTCACCATCTTTCGGGTCCCA AC	Struck et al. 2006	
H3	aF	ATGGCTCGTACCAAGCAGAC	Colgan et al. 1998	95 °C/3 min; 35 × (95 °C/40 s, 50 °C/40 s; 72 °C/1 min); 72 °C/7 min
	aR	ATATCCTTRGGCATRATRGTGAC	Colgan et al. 1998	

### ﻿Data analysis

Alignments of each gene were performed using MAFFT (Katoh et al. 2002) with default settings. The aligned and trimmed sequences were used as data sets to generate the mean genetic distances within and between the two sampling sites (Beibu Gulf and the Fujian coast) based on the Kimura’s 2-parameter (K2P) model (Kimura 1980) implemented in MEGA X (Kumar et al. 2018).

## ﻿Taxonomy

### ﻿Family Capitellidae Grube, 1862

#### ﻿Genus Promastobranchus

Taxon classificationAnimaliaCapitellidaCapitellidae

Gallardo, 1968

88F95992-4A65-5690-BF9D-019119BE3A8C

Promastobranchus Gallardo, 1968: 121; Green 2002: 324; Magalhães and Blake 2020: 391.

##### Type species.

*Promastobranchushuloti* Gallardo, 1968.

##### Diagnosis

**(after Magalhães and Blake 2020).** Prostomium rounded, without palpode; eyespots present in multiple spots. Peristomium clearly distinct from prostomium. First chaetiger biramous. Nine to thirteen thoracic chaetigers. Thoracic chaetigers with only capillaries in both rami. Thorax clearly demarked from abdomen. All abdominal segments transitional with notopodial capillaries and neuropodial hooded hooks. Branchiae absent. Genital pores, up to four pairs starting from chaetigers 9/10 or 10/11. Lateral organs present in thorax and abdomen. Pygidium adorned with a pair of ventral cirri.

#### ﻿ Promastobranchus variabilis sp. nov.

Taxon classificationAnimaliaCapitellidaCapitellidae

A8423282-1A8A-5A5E-BC83-EDD83CBD7A10

https://zoobank.org/B2610B1B-1874-4E73-9D40-C4B509CC913A

**Figure 2.**
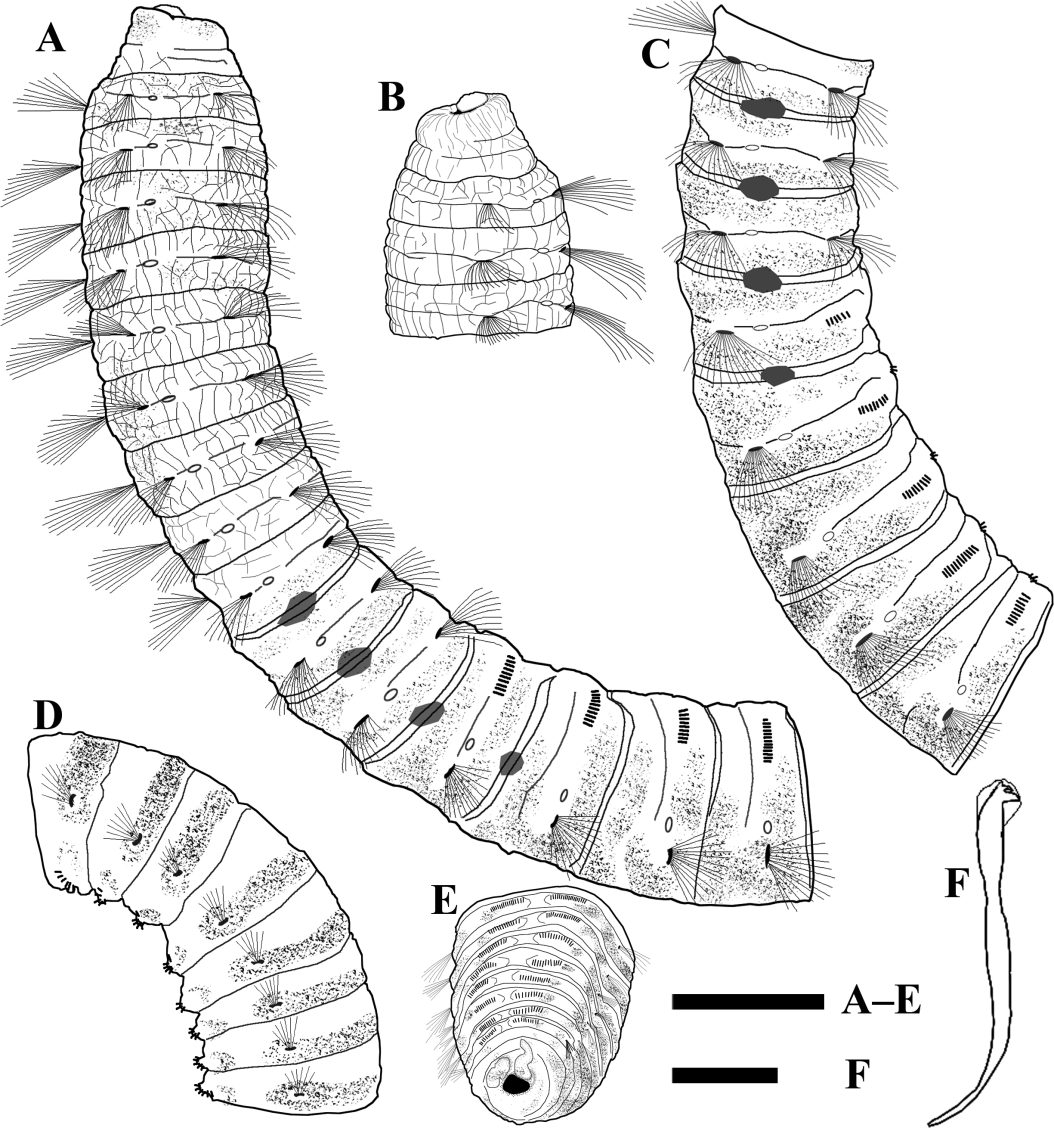
*Promastobranchusvariabilis* sp. nov., holotype **A** thorax and anterior abdomen, lateral view **B** anterior end, ventro-lateral view **C** chaetigers 9–16, lateral view **D** middle-posterior abdomen, dorso-lateral view **E** posterior end with anal cirri, ventral view **F** abdominal hooded hook of chaetiger 70. Shading on **A, C–E** indicates methyl green stain. Scale bars: 1 mm (**A–E**); 20 μm (**F**).

**Figure 3.**
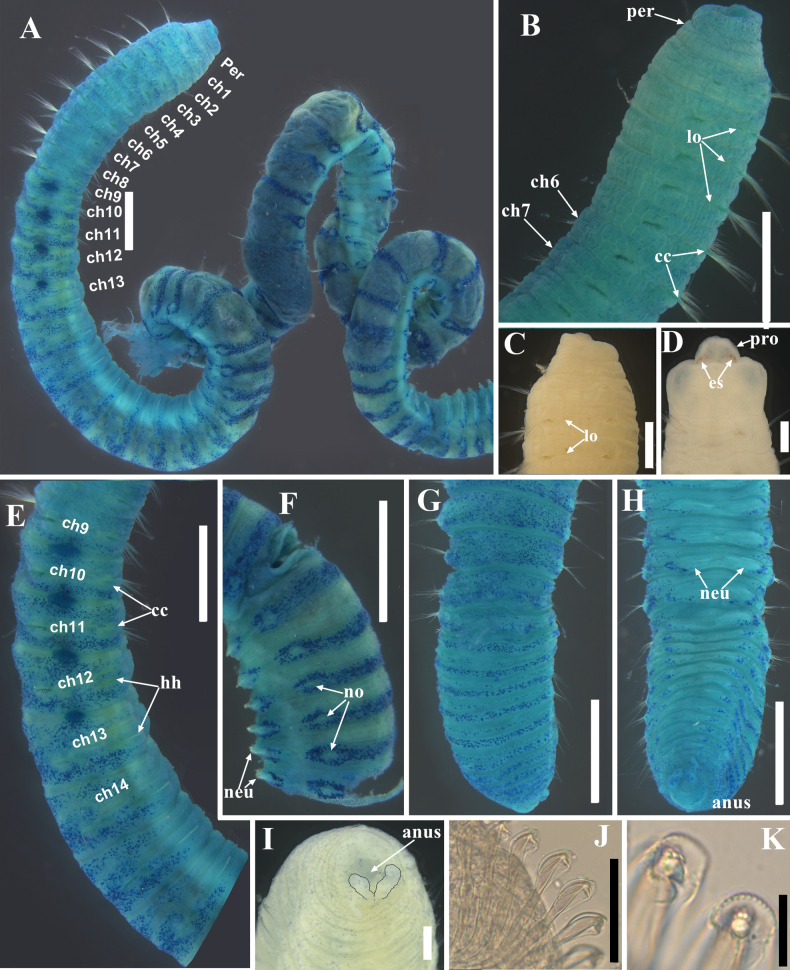
Light micrographs of *Promastobranchusvariabilis* sp. nov., holotype (**A–C, E–K**) and paratype (**D**) **A** anterior fragment showing MGSP**B** anterior thorax with areolated epithelium, ventro-lateral view **C** anterior end, lateral view **D** anterior end showing eyespots, dorsal view **E** transition between thorax and abdomen, lateral view **F** middle-posterior abdomen, dorso-lateral view **G** posterior abdomen, dorsal view **H** posterior abdomen, ventral view **I** posterior end, end view (anal cirri have been outlined with black lines) **J** hooded hooks at chaetiger 70, lateral view **K** hooded hooks, frontal view. Abbreviations: cc, capillary chaetae; ch, chaetiger; es, eyespot; hh, hooded hook; lo, lateral organ; neu, neuropodia; no, notopodia; per, peristomium; pro, prostomium. Scale bars: 1 mm (**A, B, E–H**); 0.5 mm (**C**); 0.2 mm (**D, I**); 50 μm (**J**); 10 μm (**K**).

**Figure 4.**
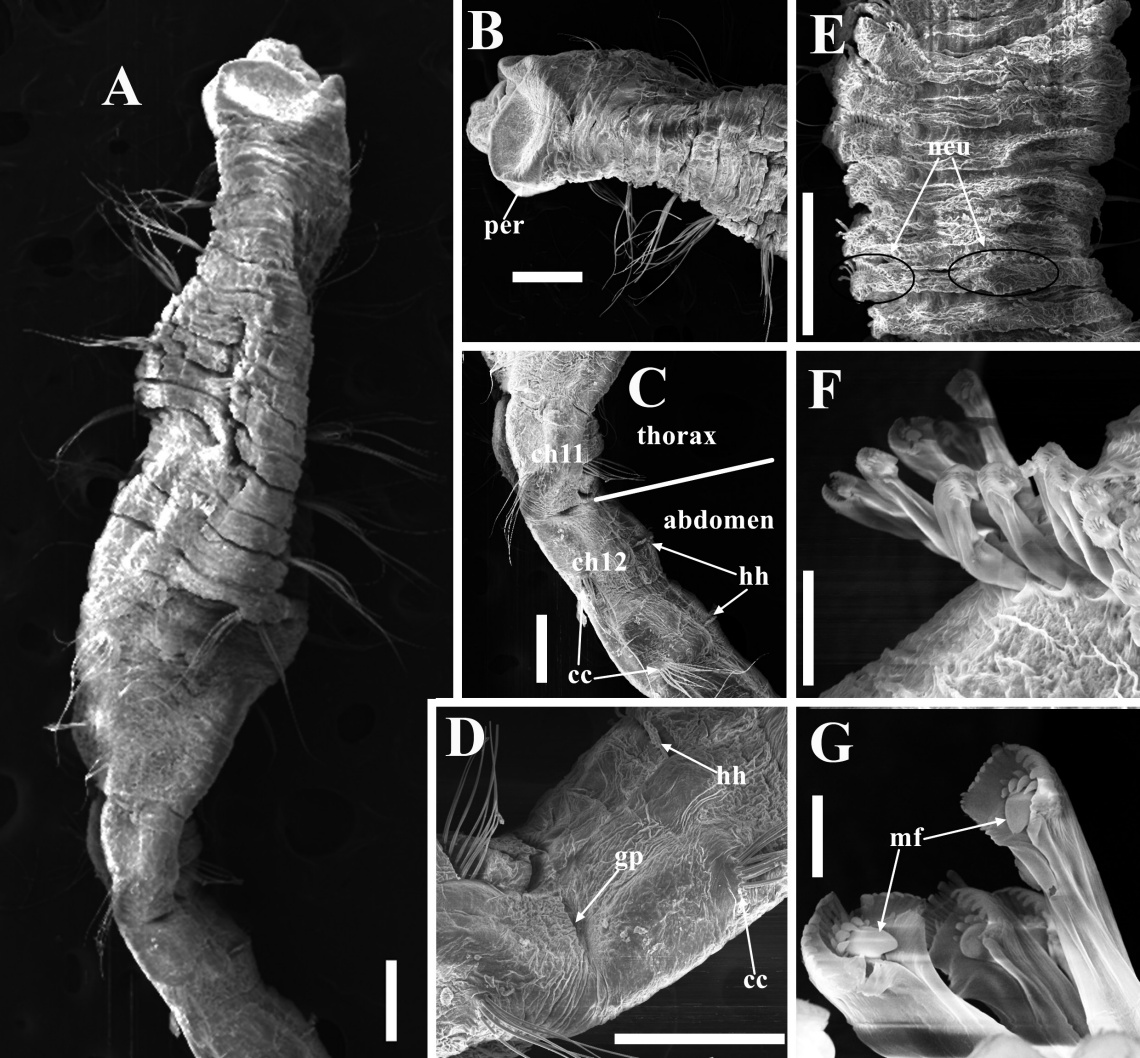
SEM photographs of *Promastobranchusvariabilis* sp. nov., paratype (TIO-BTS-Poly144) **A** anterior 13 chaetigers **B** anterior end, ventro-lateral view **C** transition between thorax and abdomen, lateral view **D** genital pore on between chaetigers 11/12, lateral view **E** posterior end showing neuropodia, ventral view **F** abdominal hooks at chaetiger 16 **G** ultrastructure of hooks. Abbreviations: cc, capillary chaetae; gp, genital pore; hh, hooded hook; mf, main fang; neu, neuropodia; per, peristomium. Scale bars: 200 μm (**A–E**); 20 μm (**F**); 5 μm (**G**).

Figs 2A–F , 3A–K , 4A–G

##### Material examined.

***Holotype***: TIO-BTS-Poly139, complete, Beibu Gulf, sta. S31 (19.65°N, 107.70°E), 65 m depth, coll. Jun-Hui Lin, 23 May 2021. ***Paratypes***: TIO-BTS-Poly140 • 1 spec., incomplete, same information as holotype. TIO-BTS-Poly141 • 4 specs, all incomplete, Beibu Gulf, sta. S42 (19.25°N, 108.10°E), 59 m depth, coll. Jun-Hui Lin, 24 May 2021. TIO-BTS-Poly142 • 3 specs, TIO-BTS-Poly143 • 5 specs and TIO-BTS-Poly144 • 6 specs, all incomplete, Beibu Gulf, sta. S44 (19.25°N, 108.50°E), 36 m depth, coll. Jun-Hui Lin, 24 May 2021. TIO-BTS-Poly145 • 4 specs, TIO-BTS-Poly146 • 3 specs and TIO-BTS-Poly147 • 1 spec., all incomplete, Beibu Gulf, sta. S51 (18.85°N, 108.50°E), 15 m depth, coll. Jun-Hui Lin, 26 May 2021. TIO-BTS-Poly148 • 1 spec., incomplete (posterior fragment with pygidium), Beibu Gulf, sta. S14 (20.456°N, 108.50°E), 55 m depth, coll. You-Ling Ye, 27 Nov 2022.

##### Description.

Holotype complete, but broken into two fragments. Anterior fragment heavily coiled (Fig. 3A), measuring 35.99 mm long by 1.27 mm wide (at chaetiger 15) for 71 chaetigers; posterior fragment measuring 10.69 mm long for 35 chaetigers. Paratypes incomplete, ranging from 2.68–19.12 mm long, 0.45–1.52 mm wide for 15–34 chaetigers. Body nearly cylindrical, widest in anterior abdomen. Color in alcohol tan (Fig. 3C). Nuchal organ not observed.

Prostomium rounded without palpode, partially concealed by peristomium (Figs 2A, B, 3A–C); eyespots present on lateral sides of prostomium when dissected (obviously seen in some specimens as shown in Fig. 3D). Proboscis retracted in holotype (Fig. 3A–C), with numerous minute papillae. Peristomium achaetous, wider than long, longer than chaetiger 1 (Figs 2A, 3A). Intersegmental groove distinct between peristomium and chaetiger 1 (Figs 2A, B, 3B, 4A, B).

Thorax with 11 chaetigers (Fig. 2A) in holotype, first chaetiger biramous (Figs 2A, B, 3B). Thoracic segments bi-annulated, wider than long, with epithelium areolated from peristomium to chaetiger 7, faintly areolated on chaetiger 8, and smooth on following segments (Figs 2A, B, 3B). Chaetigers 1–11 with only capillaries in both rami (Figs 2A, 3A), 18–30 per fascicle in notopodia and 20–30 per fascicle in neuropodia. Notopodia inserted dorso-laterally, and neuropodia ventro-lateral. Chaetal fascicles inserted just near midline of thoracic segments (Figs 2A–C, 3B). Lateral organs located between noto- and neuropodia at intrasegmental grooves, closer to notopodia in thorax and anterior abdomen, as small pores (Figs 2A, 3B). Genital pores present on intersegmental grooves of between chaetigers 9/10, 10/11, 11/12, and 12/13 in holotype.

Transition between thorax and abdomen marked by chaetal change (Figs 2A, C, 3E, 4C, D). Abdominal segments longer and wider than posterior thoracic chaetigers in anterior abdomen (Fig. 3A, E), tapering gradually towards pygidium (Fig. 3G, H). Parapodial lobes reduced in anterior abdomen (Fig. 3A, E), well separated. Neuropodial tori pad protruded above body surface from middle-posterior abdomen (Fig. 3H). Abdominal chaetigers transitional with notopodial capillaries and neuropodial hooks throughout (Figs 2C–E, 3G, H), with 15–20 notopodial capillaries and 60–70 neuropodial hooks in anterior abdomen, decreasing to 8–10 capillaries and 30–40 hooks in posterior end. In anterior abdomen, notopodial lobes located dorso-laterally and neuropodial lobes ventro-lateral (Figs 2C, 3E). From middle abdomen, notopodial lobes lateral and neuropodial lobes ventral (Figs 2D, E, 3A, H). Chaetal fascicles positioned posterior to midsegment in anterior abdomen (Fig. 3E), and near posterior edge of segment toward the pygidium (Fig. 3F). In the far posterior abdomen, neuropodial lobes close to each other with a small gap (Figs 2E, 3H, 4E).

Hooded hooks with angled node, evident constriction, developed shoulder, posterior shaft longer than anterior one, attenuated to terminal end (Figs 2F, 3J). Hood with dentate distal edge (Figs 3K, 4F, G), slightly longer than wide (Figs 2F, 3J). Hooded hooks (Fig. 4G) with three rows of teeth above main fang: four teeth in basal row, three teeth in middle row, two smaller teeth in apical row. Main fang subtriangular, longer than wide.

No branchiae observed in abdomen. Pygidium with two digitate anal cirri on ventral side (Figs 2E, 3I).

Methyl green staining pattern (Figs 2A, C, D, 3A, E–H). Body stained with pale green and small dark spots of stain scattered from peristomium to prechaetal part of chaetiger 9. Body stained dark blue in dorsum of from postchaetal part of chaetiger 9 to chaetiger 20, excluding intra- and intersegmental grooves and parapodial lobes, extending to dorsal sides of neuropodia. Four pairs of genital pores (between chaetigers 9–13) stained dark blue in holotype. From chaetiger 21, each segment has a dorsal transverse band of dark stain located on between notopodial tori, as well as stain around noto- and neuropodial tori. In far posterior abdomen, dorsum stained dark blue, reaching dorsal side of neuropodial tori, interrupted by intersegmental groove.

##### Sequences.

The amplification of the COI gene failed for all specimens. In total, 11 partial sequences of 16S, 14 partial sequences of 18S, 13 partial sequences of 28S, and 14 partial sequences of H3 were successfully obtained from 14 specimens with 9–13 thoracic chaetigers (Table 1). There is no genetic divergence (mean K2P < 0.003, with up to two base changes) among specimens from the type locality, and between the type locality and off the Fujian coast (Table 3).

**Table 3.** Mean genetic distance within and between the two sampling localities based on the K2P model. Abbreviations: BBG, Beibu Gulf; FJ, Fujian coast.

Marker	Sequence length	Within group mean distance		Between group mean distance
	bp	BBG	FJ	BBW & FJ
16S	417	0.0024	0	0.0013
18S	1694	0	0.0003	0.0002
28S	874	0.0017	0	0.0019
H3	352	0.0021	0.0014	0.0019

##### Distribution.

Currently known from Beibu Gulf (South China Sea) and off the Fujian coast.

##### Ecology.

The new species inhabits shallow-sea (10–65 m) sediments characterized by mud, muddy sand, or sandy mud with shell fragments.

##### Etymology.

The specific name was derived from its variable number of thoracic chaetigers (9–13) during ontogeny.

##### Variation.

The majority (79%) of the type specimens possess 10 or 11 thoracic chaetigers with capillaries in both rami. Larger specimens (>1.0 mm wide) have 11–13 chaetigers with only capillaries. Areolated epithelium was clearly seen in large specimens, while obscured in the small ones. The holotype stained dark blue on four pairs of genital pores (between chaetigers 9–13), whereas some specimens have blue stain on two pairs of genital pores (on chaetigers 9–11).

##### Remarks.

Among the type specimens included in this study, the holotype is the only complete one. It has more discernable morphological characters than the others, such as the form of the thoracic epithelium and genital pores and, although it was not the largest specimen, it was considered the best for the holotype. Judging from its body size (body width >1 mm), it should be a mature specimen.

The new species shares a few morphological features with the two previously known species. They all possess a rounded prostomium without a palpode, eyespots on the lateral sides of the prostomium, four pairs of genital pores on the anterior body, reduced parapodia in the anterior abdomen, two anal cirri on the ventral side of the pygidium, and a variable number of chaetigers with capillaries in both rami. Despite the highly similar body appearance, *Promastobranchusvariabilis* sp. nov. differs from *P.huloti* mainly in the dental formula of hooks and neuropodia in the preanal region, as shown in Table 4. The hooks of the new species have nine teeth in three rows (2+3+4), instead of four teeth in a single row as in *P.huloti*. The number of neuropodial hooks in the preanal region is much higher in *Promastobranchusvariabilis* sp. nov. (30–40 hooks) than in *P.huloti* (2 or 3 hooks). *Promastobranchusvariabilis* sp. nov. can be also distinguished from *P.orbiculatus* mainly by the dental formula of hooks and the position of the genital pores. The former species has nine teeth while the latter has six teeth (2+4). Besides, the new species has four genital pores present on the intersegmental grooves of chaetigers 9–13, instead of those of chaetigers 10–14 as in *P.orbiculatus*. As for MGSP, the new species has a single dark transverse band per segment located on notopodia from chaetiger 21, which was absent in the other two species. Moreover, the new species bears an areolated epithelium from the peristomium to chaetiger 8 and protruded neuropodial tori in the posterior abdomen, which are not observed in its congeners.

**Table 4.** Comparison with the known *Promastobranchus* species around the world. Abbreviations: No: notopodia; Neu: neuropodia.

Species	*P.huloti*	*P.orbiculatus*	*P.variabilis* sp. nov.
Prostomium	rounded without palpode	rounded without palpode	rounded without palpode
Eyespots	present	present	present
Thoracic epithelium	unknown	smooth	areolated up to chaetiger 8
Number of thoracic chaetigers	12–13	9–12	9–13
Branchiae	absent	absent	absent
Dental formula of abdominal hooks	4 in a single row	6 in two rows (2+4)	9 in three rows (2+3+4)
Location of genital pores	Chaetigers 9–13	Chaetigers 10–14	Chaetigers 9–13
Neuropodia in posterior abdomen	unknown	reduced	protruded above surface
Number of chaetae in preanal region	No: a few capillaries; Neu: 2–3 hooks	No: 6–8 capillaries; Neu: 30–45 hooks	No: 8–10 capillaries; Neu: 30–40 hooks
MGSP	a dorsal band of stain on chaetiger 27	dark stain around 4 pairs of genital pores	dark stain on 4 pairs of genital pores, and a dark dorsal transverse band from chaetiger 21
Habitat	8–43 m; mud, sandy mud, muddy sand, coarse sand	19–59 m; mud, sandy mud, muddy sand, sand	10–65 m; mud, sand, muddy sand with shell fragment
Type locality	South Vietnam, South China Sea	Andaman Sea, Thailand	Beibu Gulf, South China Sea
References	Gallardo 1968; Green 2002	Green 2002	This study

## ﻿Discussion

In this study, there is no genetic divergence among type specimens of *Promastobranchusvariabilis* sp. nov. with a variable number of thoracic chaetigers (9–13) using multiple gene markers, which confirmed the examined specimens belong to *Promastobranchusvariabilis* sp. nov. In other words, the new species does exhibit intraspecific variability in the number of thoracic chaetigers with capillaries. The number of thoracic chaetigers of the new species may be size dependent, and smaller individuals bear fewer thoracic chaetigers. The new species, together with the two previously known *Promastobranchus* species, possesses a variable number of thoracic chaetigers, indicating that the number of thoracic chaetigers is inappropriate to identify *Promastobranchus* species, as this character overlaps between *Promastobranchus* species. In contrast, diagnostic characters such as the dental formula of abdominal hooks, the number and location of genital pores, and the MGSP are helpful in identification, as suggested by Green (2002). The use of mature specimens is recommended for identification, as several diagnostic characters may be obscured in immature ones, such as thoracic epithelium and genital pores. In this study, we also observe the presence of an areolated epithelium in the anterior thorax and protruded neuropodial tori pad in the middle-posterior abdomen, which may be helpful in differentiating *Promastobranchus* species.

Based on genetic comparisons, the new species has a broad geographical distribution, and ranges from its type locality northeastward to the Fujian coast, which is approximately 1100 km away from the type locality. Green (2002) noted that *Promastobranchus* resembles *Scyphoproctus* in that both genera had relatively long capillary chaetae and a relatively long achaetous segment. In addition, these gerera also share a broadly rounded prostomium without a palpode and a variable number of thoracic chaetigers. Currently, the lack of molecular data of closely related genera, such as *Scyphoproctus*, hinders evaluation of the phylogenetic relationship of *Promastobranchus*.

## Supplementary Material

XML Treatment for Promastobranchus

XML Treatment for Promastobranchus variabilis

## ﻿Additional information

### Data availability

All of the data that support the findings of this study are available in the main text.
